# Vancomycin microspheres reduce postoperative spine infection in an in vivo rabbit model

**DOI:** 10.1186/s40360-016-0105-6

**Published:** 2016-11-29

**Authors:** Gang Liu, Si Chen, Jun Fang, Baoshan Xu, Shuang Li, Yonghong Hao, Naif A. Al-Dhabi, Shucai Deng, Veeramuthu Duraipandiyan

**Affiliations:** 1 Tianjin Hospital, Jiefangnan Road 406, Tianjin, 300210 China; 2 Tianjin Medical University, Qixiangtai Road 22, Tianjin, 300070 China; 3 College of bioscience and biotechnology, Hunan Agricultural University, Changsha, Hunan 410128 China; 4 Department of Botany and Microbiology, Addiriyah Chair for Environmental Studies, College of Science, King Saud University, P.O.Box.2455, Riyadh, 11451 Saudi Arabia

**Keywords:** Surgical site infections, Vancomycin microspheres, *Staphylococcus aureus*

## Abstract

**Background:**

Surgical site infections are common and devastating complications after implants related surgeries. *Staphylococcus aureus* contamination is a leading cause of surgical site infections. This study aims at assessing the effect of vancomycin microspheres on reducing *Staphylococcus aureus* infection in an in vivo rabbit model.

**Methods:**

Sixty surgical sites of 20 New Zealand White rabbits underwent spinal implant were randomly divided to three groups: the control group, the vancomycin group and vancomycin microspheres group. The surgical sites were incubated with 100 μl 1 × 10^7^ CFU *S. aureus* ATCC 25923. Prior to closure, vancomycin and vancomycin microspheres were placed into the wounds of the rabbits in the vancomycin group and the vancomycin microspheres group, respectively. The rabbits were killed on postoperative day 7. Standard quantification techniques were used to analyze biomaterial centered and soft tissue bacterial growth. The bacteria were further confirmed by PCR with primers from the thermostable nuclease gene of *S. aureus.*

**Results:**

All the rabbits survived the surgery and no postoperative wound complications or systemic illness occurred. Results showed that the bacterial cultures were 76.9, 30.8, and 15.4% in the control group, vancomycin group, and vancomycin microspheres group. Vancomycin microspheres treatments significantly decreased the infection rate compared to the control group (*p* < 0.05).

**Conclusion:**

Vancomycin microspheres combined with preoperative ceftriaxone is effective to reduce postoperative *S. aureus* infection compared with the control group.

## Background

Surgical site infections (SSIs), a common and devastating complication after implants related surgery, are substantial burden to the patients and healthcare system that works with limited budget. SSIs complicate 0.97% of all surgical cases and 21.8% of all health-care-associated infections are SSIs [1]. Each year SSIs result in about 290,000 cases in the United States. The total cost to the health care system in US is estimated to 3.3 billion dollars per year [2]. Despite the use of prophylactic antibiotics, aseptic technique, and improved surgical techniques, SSIs is a big concern especially following spine surgery. More than 100,000 dollars was spent on a single spine surgical site infection [3]. A wide variety of risk factors such as geriatric, immunocompromised, diabetic, obese and hyperglycemia, after spinal surgeries have been reported in the literature [4]. Although we have witnessed improvements in operation time, surgical techniques, and antibiotic prophylaxis in the last few years, spinal implant-related surgical site infections are still a big concern [5]. In order to control the costs and burdens to patients and the public healthcare system, it is important to discover additional techniques to reduce surgical site infections [6].

Despite meticulous sterile techniques, bacteria such as *S. aureus* contaminate the surgical wound after long procedures [7]. Hematoma, sometimes together with implants, harbored bacteria resulting in systemic infection, tissue hypoxia, and poor wound healing in patients. Patients suffer from increased back pain and higher rates of mortality [7, 8].

The use of preoperative prophylaxis for SSIs in spine implant-related surgeries has been introduced to prevent Gram-positive bacterial infections. Different antibiotics, such as cephalosporin and clindamycin, were given before and after the surgery [9, 10]. Although under ideal condition, the infections rate has been reported to be less than 1%, with the rising resistance to some common antibiotics, the infection incidence after implant-related surgeries in some countries may be higher than 10% [11] and 30–50% of *S. aureus* infections are caused by methicillin-resistant *S.aureus* (MRSA) [12]. With the emergence of MRSA, prophylactic vancomycin was introduced in spine surgery, especially for adult spinal deformity, posterior spinal fusion, and cervical spine surgery [13–16]. However, the inappropriate antibiotic concentration usually results in poor patient compliance [17].

Vancomycin-loaded poly-lactide-co-glycolide (PLGA) microspheres (Cmax: 108.19 ± 14.92 ng/ml at tmax of 1.33 ± 0.58 h, the t1/2: 120.65 ± 44.18 h [18]) was designed to deliver near-linear level of antibiotic agent for at least 4 weeks [19]. It has an advantage of providing high local concentrations of antibiotics for a prolonged period after surgery and avoiding the risk of systemic toxicity over intravenous administration [20, 21]. Gilchrist et al. showed fusidic acid and rifampicin co-loaded PLGA microspheres showed direct antimicrobial activity against *S.epidermidisin vitro* [22]. To date, there is limited evidence of vancomycin-loaded microspheres in reducing Gram-positive bacteria causedinfections, particularly *S. aureus* infections. In this study, to evaluate the efficiency of vancomycin-loaded microspheres in preventing the implant-related spinal surgeries, we analyzed infection rates and bacterial cultureafter the surgeries in a well-known New Zealand White rabbits spinal implant model.

## Methods

### Bacterial preparation

*Staphylococcus aureus* (ATCC 25923), which is sensitive to vancomycin (MIC: 0.9 μg/ml), but not sensitive to ceftriaxone, was used in this study [23]. *S. aureus* was cultured on trypticase soy agar (Oxoid item number LP0042), transferred to trypticase soy broth. The bacteriawere incubated for 12 h at 37 °C. After centrifuging at 2,000 g for 5 min, the pellets wereresuspended and diluted to different levels, the concentration of the bacteria was determined at 550 nm with a spectrophotometer (UV1600, Mapada Equipment Co. Ltd., Shanghai, China) and further estimated by plating on trypticase soy agar plates. Based on the previous report, 100 μl 1 × 10^7^ CFU/mL of *S. aureus* ATCC 25923 was used to create a reliable infection rate [24].

### Experiment design

Twenty New Zealand White female rabbits with a body weight of 3.8 ± 0.21 were used in the surgeries. Completely randomized block design in this research, each rabbit was considered as a block. In each rabbit, three surgical sites, T13, L3, and L6, were randomly allotted to each of the three treatments: the control group, the vancomycin group and vancomycin microspheres group. To mimic preoperative prophylaxis, all the groups received a preoperative 20 mg/kg ceftriaxone according to a previous study [24]. Prior to closure, 150 mg microspheres (PLGA), 50 mg vancomycin, and 200 mg vancomycin microspheres (PLGA:vancomycin, 75:25, resorption in 3–7 days) were locally delivered to the control group, the vancomycin group, and vancomycin microspheres group, respectively.

### Surgical procedure

The surgical procedure was slightly modified according to Poelstra et al. [25]. The entire back and major parts of the animals’ gluteal region were thoroughly shaved 1 day before the surgery. After being fasted for 12 h, animals were injected intramuscularly with a combination of 5 mg/kg xylazineand 44 mg/kg ketamine. During the surgery, anesthesia was maintained by using isoflurane inhalation via nose-cone mask.

Three non-continual sites (T13, L3, and L6) were marked on the back of the animal. The surgical approach was the same for each site. After the back was sterilized, a 2-cm dorsal skin incision was made longitudinally in the midline, followed by a single incision in the fascia to expose the spinous process. Using a small rongeur, the entire spinous process with surrounding musculature and ligaments was excised from the base (weighing 0.08–0.10 g) to create a hollow self-contained defect, approximating a partial laminectomy defect. The ligamentumflavum was not violated, and the dura was not exposed. An 0.85-mm diameter stainless steel rod (2-mm diameter, Item: TI017905, Goodfellow corporation, Oakdale, PA) was implanted into the defect from the left side of the rabbit. A 100 ul 1 × 10^7^ CFU *S. aureus* was injected inside the defect pocket and onto the implant to create a 70% infection rate [24]. After mixed with a flowable hemostatic agent (Integra LifeSciences Corporation, Plainsboro, NJ), combined with a 150 mg microspheres, a 50 mg vancomycin, or a 200 mg vancomycin microspheres (assigned randomly to T13, L3, or L6 by using a random number generator), the wound was closed using running sutures with biodegradable Vicryl 2/0 suture (Ethicon Inc. Piscataway, NJ) and a running subcutaneous suture with Vicryl 3/0 (Ethicon Inc.). The second implantation was performed with the same procedure at the next randomly selected site and the same procedure was repeated at the last site except the treatment is different. To prevent cross-contamination, different sterile instruments and drapes were used for each surgical site. After the surgery, the animals were housed individually and permitted to drink and eat ad libitum in standard cages equipped with water and standard antibiotic-free rabbit chow. The rabbits were monitored daily, with particular attention to wound healing, temperature, body weight, and signs of sepsis.

The body weights of the animals were measured before the surgeries and body temperatures were measured at 9:00 each day. One animal per time point were selected randomly, weighted and killed at 12, 24, 48, 72, 96, 120, and 144 h to determine vancomycin concentration and the other 13 animals were weighted and killed at 168 h. All the rabbits were killedvia a 10-mg/kg intravenous pentobarbital injection according to the approved protocol. A 2 × 2-cm right liver lobe and 5 ml intravenous blood were collected to monitor systemic infection. Systemic infection was defined as body temperature goes high, food and water intake drops, and the pathogen is distributed throughout the body. Samples of the fascia, the hematomaand the vertebral lamina were harvested. The implanted metal rods were removed. Surgical site infection was defined as redness around the surgical area and drainage of cloudy fluid from the surgical wound.

### Vancomycin concentration analysis

After hematoma samples were harvested from the implant sites, vancomycin, released vancomycin and vancomycin microspheres were determined according to Burcu S. et al [26]. In short, the hematoma was suspended in pH 7.4 phosphate buffer and kept in an ultrasonic bath for 5 min. After centrifugation, the supernatant, representing the vancomycin and released vancomycin from vancomycin microspheres, was detected by a spectrophotometer (UV1600, Mapada Equipment Co. Ltd., Shanghai, China)at 280 nm. Meanwhile, 10 ml pH 7.4 phosphate buffer was added after the precipitate was dissolved in 3 ml methylene chloride. The polymer was totally removed after the evaporation of methylene chloride, the solution was filtered and vancomycin content representing vancomycin microspheres was detected by a spectrophotometer at 280 nm.

### Bacterial evaluation

All samples were evaluated by a team member who is blind to the treatments. Harvested tissue samples were immediately weighed and homogenized (Roche MagNALyser), the implants were sonicated (Sonics VCX-130-PB, Newtown, CT) for 15 min to detach bacteria at 4 °C. Serial dilution samples were plated and incubated on trypticase soy agar plates for 24 h at 37 °C. The bacteria were further confirmed by PCR with primers (5′-GCGATTGATGGTGATACGGTI-3′) and (5′-AGCCAAGCCTTGACGAACTAAAGC-3′), which came from the thermostable nuclease gene of *S. aureus* [27]. The final CFU of *S. aureus* was determined per gram of tissue samples and per centimeter of stainless rod at every site.

### Statistical analysis

Sample size analysis was performed according to the instruction of Infostat 2013. When the power function is higher than 0.80, the sample size is considered correct. All the other statistical analyses were performed by using the SPSS 20.0 software (Chicago, IL, USA). Data were presented as means and standard deviation (SD). Chi square (χ2 calculations) were used to determine whether there are significant differences in infection rates. One-way analysis of variance (One-way ANOVA) was used to determine whether significant variation existed among different treatments. Differences between means were determined by a LSD (Least Significant Difference) test while overall differences were found. All differences at a *P* < 0.05 level were considered significant.

## Results

There was no difference in duration of surgery. The rabbits had similar body weight before and after surgery. All the rabbits survived the surgery and no postoperative wound complications or systemic illness occurred. Vital functions such as temperature, food, and water intake also showed that no systemic infection occurred. The power functions for infection rates and bacterial culture analysis are 0.998 and 0.968 respectively, which indicate the sample size is suitable for this research.

The infection rate was evaluated after 7 days of the surgery. 10 SSIs and seven implant infections out of 13 infection sites were found in the control group, with a 76.9 and 61.5% infection rate, respectively. In vancomycin group, 4 SSIs and two implant infections out of 13 infection sites were found. Meanwhile, in vancomycin microspheres group. 2 SSIs and 0 implant infections out of 13 infection sites showed evidence of infections (Table 1). The incidence of infection was significantly reduced in vancomycin microspheres groups compared with the microspheres control group (*p* < 0.05). Although the incidence of SSIs decreased by 60% in vancomycin group (4 infections out of 13) compared with the control group (10 infections out of 13), no significant difference was detected between these two groups. Meanwhile, there was no significant difference in the infection rate between vancomycin group and vancomycin microspheres group.

**Table 1:** Postoperative surgical site infection and implant-related infection incidence in the control, vancomycin, and vancomycin microspheres groups

Treatment (*n* = 13)	Total Sites	Surgical site infection	Implant infection	Non-infection
Control	13	10(76.9%)^a^	8(61.5%)^a^	3(23.1%)^a^
Vancomycin	13	4(30.8%)^ab^	2(15.4%)^ab^	9(69.2%)^ab^
Vancomycin microspheres	13	2(15.4%)^b^	0(0.0%)^b^	11(84.6%)^b^
*P* value		0.004	<0.001	0.004

^ab^Means with different letters within a column differ significantly (*P* < 0.05)

Bacteriologic colony counts were showed in Table 2. For fascia, hematoma, and implant bacteriologic colonies, there was no significant difference between three groups. Whereas in bone samples, bacteriologic colonies were significantly greater in the vancomycin group than the other groups (*p* < 0.05). For blood and liver samples, no bacterial growth was detected.

**Table 2:** Bacterial culture analysis of different samples in Log10 values

(Log_10_ CFU/g tissue)	Control		Vancomycin		Vancomycin microspheres		F	*P* value
	mean	sd	mean	sd	mean	sd		
Fascia	5.810	0.285	6.067	0.208	6.150	0.212	2.012	0.176
Hematoma	6.820	0.244	7.075	0.320	6.650	0.354	1.940	0.183
Implant	5.525	0.328	5.450	0.212			0.090	0.772
Bone	5.500^a^	0.283	6.233^b^	0.252	5.800		7.749	0.011

^ab^Means with different letters within a row differ significantly (*P* < 0.05)

**Fig. 1:**
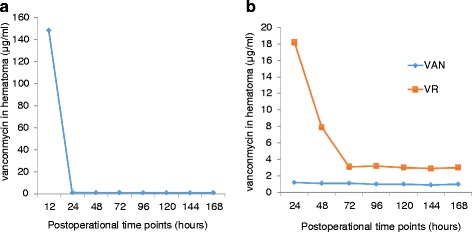
Vancomycin concentration in vancomycin and vancomycin microsphere groups. **a** Local vancomycin in vancomycin group was highest at 12 h with a concentration of 148 μg/ml and dropped to 1.2 μg/ml quickly at 24 h. **b** In vancomycin microsphere group, the release of vancomycin (VR) was highest at 24 h and maintained a concentration of 3.0 μg/ml after 72 h. The concentration of vancomycin remaining in the microsphere (VM) dropped constantly

In vancomycin group, local vancomycin was found to be the highest at 12 h with a concentration of 148 μg/ml. Whereas, it dropped to 1.2 μg/ml quickly at 24 h (Fig. 1a). In vancomycin microspheres group, The concentration of vancomycin remainingin the microsphere (VM) dropped constantly. The release of vancomycin was highest at 24 h. After dropped to 3.2 μg/ml at 72 h, it maintained a concentration of 3.0 μg/ml (Fig. 1b).

## Discussion

Surgical site infections are the most common postoperative infections in spinal surgeries. In order to reduce SSIs, the use of systemic antibiotic prophylaxis has become common practice and successfully lowered the risk of infection [28, 29].

Systemic antibiotic prophylaxis, including cefazolin and vancomycin, rely heavily on diffusion into the surgical wound [30]. The concentration of the antibiotics around and within the wound may not reach the MIC of the antibiotics [30]. Various studies showed that vancomycin is effective to fight against postoperative *S. aureus* infections [11, 12]. Locally delivered antibiotics have an advantage over preoperative antibiotics for they offer a high concentration at the wounds [24, 31]. Locally administered antibiotics can reach levels twenty times of the toxic levels while maintaining a safe systemic concentration. Meanwhile, there are also concerns about the high concentration applied locally may be cytotoxic. Bosso et al. reported that a concentration of > 15 μg/ml vancomycin was related with a 3-fold increased risk of nephrotoxicity [32].

In our study, three surgical sites, T13, L3, and L6, are at least 4 cm from each other, so the risk of diffusion of antibiotics or cross-infection between sites is low. The results showed that vancomycin microspheres were effective to reduce the bacterial infections. Usually, 1 to 2 g vancomycin was used in spinal surgery. Sweet et al. showed 2 g vancomycin powder was used without systemic toxicity [33]. Considering the average rabbit body weight of 3.8 kg, the dose of 50 mg vancomycin or 200 mg vancomycin microspheres in our study would be about the same to 1 g vancomycin for an 80-kg patient.

According to Stall et al., 10^6^ CFU per site were sufficient to produce a 70% infection rate. In their study, five out seven sites were infected, with an infection rate of 71% [24]. In a previous intrawoundvancomycin study with the same bacteria, Lukas et al. showed the infection rate was 100% with 10^7^ CFU per site [33]. For the fact that *S. aureus* could not reach such a high level of 10^7^ in patient’s surgery; we used the same bacteria and a low bacterial concentration (10^6^ CFU per site) in our study. The results showed a 76.9% infection rate, which was similar to Stall’s reports [24].

Different from a previous report by Lukas et al., in which they showed that 100 mg intrawoundvancomycinpodwer eliminated all the *S. aureus* infections [33], our results showed that 50 mg vancomycin and 200 mg vancomycin microspheres were not sufficient enough to eliminate all the infections. This may be due to a lower concentration of vancomycin used in our study. Stall et al. showed that 2.5 mg gentamicin microspheres produce a 38% infection rate, which decreased significantly compared with the microspheres control group [24]. Although PLGA was biodegradable and safe, Lukas et al. pointed out that microspheres could act as a foreign body for bacterial adhesion, which may cause a bacterial infection in Stall’s study [33]. While Stall et al. did not show the infection rate in gentamicin control group, our results showed that vancomycin microspheres treatment (15.4% infection rate), better than vancomycin treatment (30.8% infection rate), resulted in a significant decrease in infection rate compared with the control group.

In an earlier study, Yenice et al. showed that teicoplanin loaded biodegradable microparticles PLGA (75:25) polymer were the most effective and promising for obtaining prolonged local antibiotic release and fighting against staphylococci infection [34]. Burcu et al. reported vancomycin-loaded PLGA microspheres provided a controlled antibiotic release and seemed to be a promising carrier system for antibiotic delivery [26]. For this reason, a PLGA copolymer was used in this study to obtain a prolonged delivery of vancomycin. Although MIC creep, a process with a sustained increase in the MICs of glycopeptides against *S. aureus*, is a big concern to the prolonged exposure to vancomycin, Joana et al. reported that no MIC creep was found in an over 3-year study in a tertiary hospital in Portugal [32]. Further study should focus on the toxicity and MICs before and post antibiotic exposures.

There are increasing reports of *S. aureus* strains showing resistance to 1–4 mg/ml vancomycin [35, 36]. Several authors pointed out that the vancomycin MICs for the strains are not stable [37, 38]. In our study, 12 h after the surgery, local vancomycin with a concentration of 148 μg/ml was found to be the highest in vancomycin group. It dropped to 1.2 μg/ml quickly after 24 h, which may notbe effective to eliminate all the bacteria. This may partly explain why there were four infections in the vancomycin group. In vancomycin microspheres group, the release of vancomycin was highest at 24 h. Afterwards,it dropped to3.2 μg/ml at 72 hoursand maintained a concentration of 4.8 μg/ml until 168 h.

## Conclusion

The results of this study in a well-established New Zealand White rabbit model demonstrated vancomycin microspheres significantly decreased the incidence of implant-associated postoperative infections compared with the control group. The combination of antibiotic-loaded microspheres provides a controlled drug delivery system for infections.

## Abbreviations

CFU: Colony-forming units
MIC: Minimum inhibitory concentration
PLGA: Poly-lactide-co-glycolide
SSIs: Surgical site infections
